# Supplementary material for: A journey through the Corynebacterium pseudotuberculosis proteome promotes insights into its functional genome
Source: PeerJ. 2021 Dec 23;9:e12456. doi: 10.7717/peerj.12456 (PMC8710256; doi:10.7717/peerj.12456)
Supplement: Supplemental Information 1 [file peerj-09-12456-s001.pdf]

Table S.1: List of host proteins detected exclusively in diseased lymph nodes from the study conducted by Rees et al. (2015a)

Protein	Biological process and/or Molecular function
Cathelicidin-2	Defense response to bacterium
Complement component C3 (Fragment)	Complement activation
Regakine 1-like protein	Immune response
CD11b	Integrin-mediated signaling pathway
MHC class I antigen	Antigen processing and presentation/immune response
MHC class I antigen	Antigen processing and presentation/immune response
Fc gamma 2 receptor	Immune response
Osteopontin	Biomineral tissue development
Lactoferrin	Immune response/antimicrobial activity
Alpha-2-HS-glycoprotein	Regulation of inflammatory response
Serum amyloid A protein (fragment)	Inflammatory response
SERPINF1	Regulation of neurogenesis and angiogenesis
Serpin peptidase inhibitor clade B ovalbumin member 1	Negative regulation of interleukin-1 beta production
175 antigen (Fragment)	Inflammatory response
Heat shock protein 70	Stress response
Alpha-1-antitrypsin transcript variant 1	Serine-type endopeptidase inhibitor activity
Chitinase-3-like protein 1	Inflammatory response
Cytochrome b-245 alpha polypeptide (fragment)	Heme binding
Cytochrome b-245 beta polypeptide (fragment)	Oxidoreductase activity
Fructose-1,6-bisphosphatase 1	Carbohydrate metabolism
Putative H-ATPase subunit B (Fragment)	Ion transport
Pyridoxal kinase	Pyridoxal 5'-phosphate salvage
Coagulation factor II	Blood coagulation/acute-phase response
Protein-arginine deiminase type-3	Protein citrullination
SRI	Transcriptional regulation
LDHA protein (fragment)	Carbohydrate metabolism
Ceruloplasmin	Copper ion transport
Lactate dehydrogenase A (fragment)	Carboxylic acid metabolic process
Histone H2A.Z	Cellular response to estradiol stimulus
Insulin-like growth factor-binding protein-6	Insulin-like growth factor binding
Solute carrier family 2, facilitated glucose transporter member 3	Glucose transmembrane transport
Enolase (Fragment)	Glycolytic process
RAB10	GTP binding

14-3-3 protein zeta/delta	Signal transduction
NADPH oxidase heavy chain subunit (fragment)	Oxidoreductase activity
Serum albumin	Metal ion binding
Carbonic anhydrase 2	Angiotensin-activated signaling pathway/ dipeptide Transmembrane transport
Angiotensinogen	Vasoconstriction
Ubiquitin-60S ribosomal protein L40	Translation
Asparaginase-like 1 protein (fragment)	Hydrolase activity
Beta-1,4-galactosyltransferase I	Protein glycosylation
Carnitine	Carnitine O-palmitoyltransferase activity
Copine I	Copine-domain
Enolase 1 (fragment)	Glycolytic process
Eukaryotic translation initiation factor 5A	Translation
Protein kinase C inhibitor KCIP-1 isoform eta (fragment)	Unknown function
RHOA	Signal transduction
Seryl-tRNA synthetase (fragment)	Selenocysteinyl-tRNA(Sec) biosynthetic process
Vimentin (fragment)	Stabilization of type I collagen mRNA
